# Supplementary material for: Understanding stroke survivors’ preferences regarding wearable sensor feedback on functional movement: a mixed-methods study
Source: J Neuroeng Rehabil. 2023 Nov 1;20:146. doi: 10.1186/s12984-023-01271-z (PMC10621082; doi:10.1186/s12984-023-01271-z)
Supplement: Supplementary file 1 — Additional file 1: Interview guide. [file 12984_2023_1271_MOESM1_ESM.pdf]

Supplementary material

Interview guide

1. **Overall experience (results from this section reported in another manuscript)**

- Do you think the previous week was representative of your typical everyday activities?
    - How was it different than usual?
    - Do you think wearing the MiGo sensors made you move more or less than what you would consider your 'normal' amount of activity?
- Please describe your experience wearing the MiGo sensors.
- How comfortable was each sensor?
- Tell me the problems you experienced during the study (i.e., technical issues, injury).
- What do you feel are some advantages of using the MiGo for rehabilitation?
- What do you feel are some disadvantages of using the MiGo for rehabilitation?

2. **Perception of the Movement report**

A. Let's look at the graphs that represent your arm movement.

- Tell me which graphs do you like the best?
    - Are there any graphs that are confusing to you?
- Which graph motivates you to move your affected arm more?

B. Now, let's look at the graphs that represent how much and how well you walk.

- Tell me which graphs do you like the best?
    - Are there any graphs that are confusing to you?
- Which graph motivates you to increase the amount you walk in your home?
    - In the community?
- Which graph motivates you to improve the quality of how you walk in your home?
    - in the community?

C. Feedback delivery

- How would you like the Movement report to be offered? (e.g., on an app on your phone, by email, on the watch itself, during a meeting with a clinician)
- How would you use this Movement report?
    - If feedback was offered on the watch or on an app on your phone, would you use it?
- How useful do you find this type of feedback to increase/improve the movements of your arm/walking?

D. How can the Movement Report be improved?

E. Is it helpful to receive objective feedback (like the Movement Report) on your activity?

- How often would you prefer to receive this feedback?
    - More than once per day, daily, weekly, or less than once per week?

- Now that we have discussed the Movement Report, do you feel that you could navigate and use this information on your own (or with help from a caregiver)?
- How would you prefer to receive feedback? (e.g., delivered through the Movement Report? by speaking with someone directly? a combination of both? or a different method?
